# Human *Borrelia miyamotoi* Infection, Austria

**DOI:** 10.3201/eid2609.191501

**Published:** 2020-09

**Authors:** Selma Tobudic, Heinz Burgmann, Gerold Stanek, Stefan Winkler, Anna-Margarita Schötta, Markus Obermüller, Mateusz Markowicz, Heimo Lagler

**Affiliations:** Division of Infectious Diseases and Tropical Medicine, Medical University of Vienna, Vienna, Austria (S. Tobudic, H. Burgmann_,_ S. Winkler, M. Obermüller, H. Lagler);; Institute for Hygiene and Applied Immunology, Center for Pathophysiology, Infectiology and Immunology, Medical University of Vienna, Vienna (G. Stanek, A.-M. Schötta, M. Markowicz)

**Keywords:** recurrent fever, Borrelia miyamotoi, rituximab, tickborne infections, vector-borne infections, bacteria, Austria

## Abstract

We report a human case of *Borrelia miyamotoi* infection diagnosed in Austria. Spirochetes were detected in Giemsa-stained blood smears. The presence of *B. miyamotoi* in the patient’s blood was confirmed by PCR, and phylogenetic analysis identified an infection with a strain from Europe.

*Borrelia miyamotoi* is a relapsing fever spirochete transmitted by the same genus of ticks that transmits *B. burgdorferi* sensu lato (s.l.), *Anaplasma phagocytophilum*, *Babesia* species, and tickborne flaviviruses (*1*–*3*). *B. miyamotoi* has been documented in ticks from the United States and in numerous countries in Europe (including Russia), as well as in Japan (*1*,*4*–*6*). *B. miyamotoi* also has been found in *Ixodes scapularis* ticks in the northeastern and north-central United States and adjoining areas of Canada, in *I. pacificus* ticks in the far western United States and British Columbia, in *I. ricinus* ticks in Europe, and in *I. persulcatus* ticks in Europe and Asia (*1*,*7*,*8*). *I. pavlovskyi* and *I. ovatus* ticks in northern Asia are 2 other species that have been shown to carry *B. miyamotoi* (*9*). Endemic areas of *B. miyamotoi* in *Ixodes* ticks overlap with those of *B. burgdorferi* s.l. but with 10-fold lower prevalence (*4*). Co-infection of *Ixodes* ticks with both spirochetes also has been identified (*9*).

Unlike Lyme borreliosis, patients with *B. miyamotoi* disease typically do not have skin lesions but instead have a nonspecific febrile illness, potentially associated with leukopenia, thrombocytopenia, and elevated liver function parameters (*10*). Highly immunocompromised patients might have chronic meningitis (*2*). Untreated patients with *B. miyamotoi* disease might experience a limited number of recurrent episodes of fever, similar to other relapsing fevers caused by *Borrelia* infections (*6*). The same antibiotic regimens used to treat Lyme borreliosis (e.g., 10−14-day courses of oral doxycycline or amoxicillin) are effective for *B. miyamotoi* disease. Parenteral therapy with ceftriaxone would be preferred for patients with meningitis (*3*).

Diagnosis of *B. miyamotoi* disease should be considered in any patient who has fever attacks and resides in or has spent time during tick season in a region where Lyme borreliosis is endemic. Diagnosis requires confirmation using PCR. If the density of spirochetes in the blood is >10^4^/mL, spirochetes might be identified by examining several high-power fields of the blood smear or centifuged sample of cerebrospinal fluid stained with Giemsa or Wright stain (*10*). Several PCR assays can detect *B. miyamotoi* in whole blood, plasma, and cerebrospinal fluid by using primers specific for 16S ribosomal RNA and for the *flaB* and *glpQ* genes (*2*,*3*,*8*). Serologic testing based on glycerophosphodiester phosphodiesterase antigen of *B. miyamotoi* that is not found in *B. burgdorferi* s.l. is highly sensitive but only on convalescent-phase serum specimens (*11*).

## The Case-Patient

A 51-year-old woman who had a long medical history of seropositive rheumatoid arthritis treated with rituximab sought care at our outpatient clinic for relapsing fever that started 3 months before. Fever episodes occurred every 5 days, and duration ranged from 2 to 3 days. Four weeks before the onset of symptoms, the patient had returned from a 3-week trip through the United States, where, as a tourist, she visited the East and West Coasts and stayed in several national parks. She reported several insect bites and 1 tick bite without erythema migrans that occurred while she was in the United States but did not notice any tick bites before or after her travel. After her return, the patient did not travel abroad again but spent her time in her home in lower Austria. 

**Figure 1:**
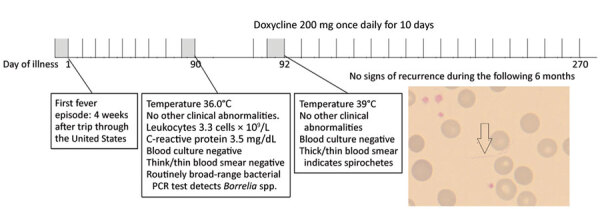
Timeline of the course of symptoms and treatment, including laboratory test results, for a patient with *Borrelia miyamotoi* infection (including Giemsa stain of thin blood smear on day 92), Austria. Arrows indicate spirochetes. Original magnification × 100.

**Figure 2:**
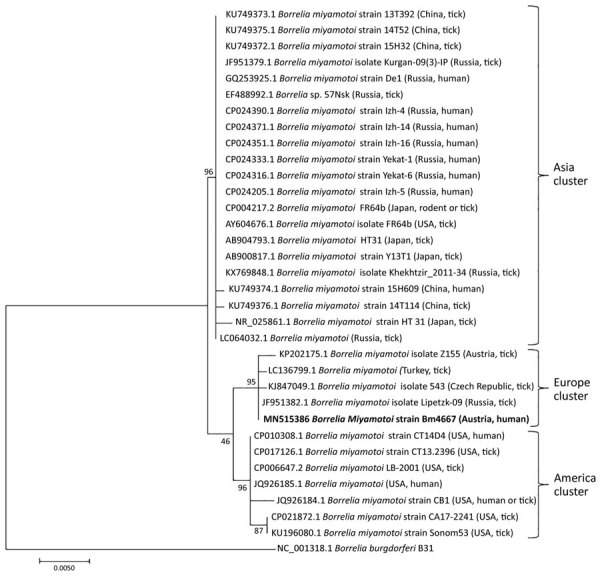
Phylogenetic tree based on the 16S rRNA gene of *Borrelia miyamotoi* from a patient in Austria (Bm4667; arrow) and reference sequences. This phylogenetic tree was constructed by using the maximum-likelihood method based on the general time reversible model. The tree is drawn to scale, with bootstrap values shown at the nodes of the tree, inferred from 600 replicates. A total of 1,199 bp of *B. miyamotoi* 16S rRNA gene sequences was used in the final dataset, which involved 34 nt sequences and in which gaps and missing data were deleted. Evolutionary analyses were conducted in MEGA7 (https://www.megasoftware.net). The patient isolate clustered together with strains found in ticks from Europe. Information of the source and country for reference isolates is shown in parentheses after strain designation. Scale bar indicates substitutions per site.

No abnormal findings were observed on physical examination; in particular, no apparent rash on the skin was noted. Routine laboratory tests performed were normal (including kidney and liver function tests), except for the evidence of leukopenia with 3.3 × 10^9^ cells/L (reference range 4–10 × 10^9^ cells/L) and slightly elevated C-reactive protein of 3.5 mg/dL (reference range <0.5 mg/dL). Multiple blood and urine cultures were negative (Figure 1). An initial peripheral Giemsa-stained blood smear was performed during an afebrile period without any result, but when the test was repeated during the next fever episode, spirochetes were detected between blood cells (Figure 1). Detection of spirochetes in blood smear, which is not typical in cases of *B. miyamotoi* infection, could be attributable to prolonged spirochetemia likely associated with rituximab therapy. *Borrelia* spp. was identified in a routinely broad-range bacterial 16S gene-based PCR test (SepsiTest-UMD; Molzym GmbH & Co. KG, https://www.molzym.com). DNA was extracted from EDTA blood, and a real-time PCR assay specific for *B. miyamotoi* targeting the *glpQ* gene (*5*) was positive. To confirm the genospecies present in the patient’s blood, further PCRs targeting the 16S–23S internal transcribed spacer region (*12*,*13*), 16S rRNA, and *glpQ* gene (*14*,*15*) were performed, and amplicons were sent to a laboratory for bidirectional sequencing (Microsynth, Vienna, Austria). All PCRs confirmed the presence of *B. miyamotoi* in the patient’s blood, and all yielded 100% identity to various *B. miyamotoi* strains in the GenBank database. Using MEGA7 (https://www.megasoftware.net), we constructed a phylogenetic tree for our isolate Bm4667 on the basis of the obtained 16S rRNA gene sequence (Figure 2). All 3 sequences obtained during this investigation were submitted to GenBank (accession no. MN515386 for the 16S rRNA gene, MT396940 for the *glpQ* gene, and MT396941 for the 16S–23S internal transcribed spacer region).

The patient was treated with 200 mg doxycycline once daily for 2 weeks. On the first day of antibiotic administration, the patient was admitted to our hospital for observation in case of potential Jarisch–Herxheimer reaction. However, during the therapy, no reaction or adverse effects were detected. The patient recovered successfully, and no signs of recurrence were observed in the following 6 months. 

## Conclusions

We describe a human case of *B. miyamotoi* infection diagnosed in Austria. Although the patient’s report of a tick bite in the United States suggested that this infection was an imported case, the phylogenetic analysis of the *B. miyamotoi* strain clearly indicates an infection with a strain from Europe (and possibly Austria). Clinicians should be aware of the possibility of these infections.
